# A chronic myeloid leukemia present with an unusual relapse in central nervous system: A case report

**DOI:** 10.1002/ccr3.8671

**Published:** 2024-03-26

**Authors:** Mais Musleh, Qossay Alhussien

**Affiliations:** ^1^ Department of Hematology, Faculty of Medicine Al Assad University Hospital Damascus Syria; ^2^ Department of Hematology, Faculty of Medicine Al‐Mouwasat University Hospital Damascus Syria

**Keywords:** blast crisis, central nervous system, chronic myeloid leukemia, tyrosine kinase inhibitor

## Abstract

Extramedullary involvement of the central nervous system (CNS) in Chronic Myeloid Leukemia (CML) is an uncommon relapse. In this case, we present a unique instance of a 43‐year‐old male diagnosed with CML experiencing a CNS blast crisis.

## Graphical abstract

Blast cell in CSF examination.

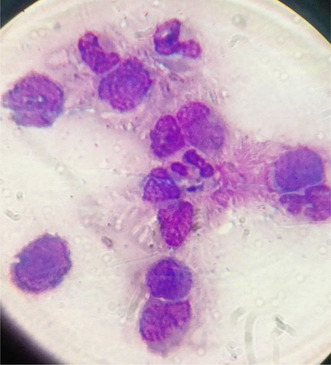

## 1 INTRODUCTION

Chronic myeloid leukemia (CML) is a myeloproliferative disorder characterized by increased proliferation of granulocytes with retained differentiation potential. Diagnostic confirmation depends on the identification of the Philadelphia chromosome arising from a reciprocal translocation t(9;22) (q34:q11) involving chromosomes 9 and 22. This translocation culminates in fusion of the breakpoint cluster region (BCR) with the ABL gene, giving rise to an oncoprotein endowed with tyrosine kinase functionality.^1^, ^2^ CML is typically diagnosed in the chronic phase (CP), although it is less commonly present as an accelerated crisis or even progresses to acute leukemia, known as a blast crisis.^3^ The initial treatment for CML is tyrosine kinase inhibitors (TKIs) such as imatinib, which achieve deep molecular responses in the majority of cases.^4^, ^5^ However, CML rarely manifests as a central nervous system (CNS) blast crisis, typically observed in patients treated with imatinib.^6^, ^7^, ^8^, ^9^, ^10^, ^11^, ^12^, ^13^ This occurrence can be attributed to the limited permeation of the drug through the blood–brain barrier, designating the CNS as a sanctuary locale.^14^, ^15^ This report details the extremely rare case of a 43‐year‐old male CML patient experiencing an extramedullary CNS blast crisis recurrence and provides treatment options and strategies for disease response monitoring.

## 2 CASE HISTORY

**FIGURE 1:**
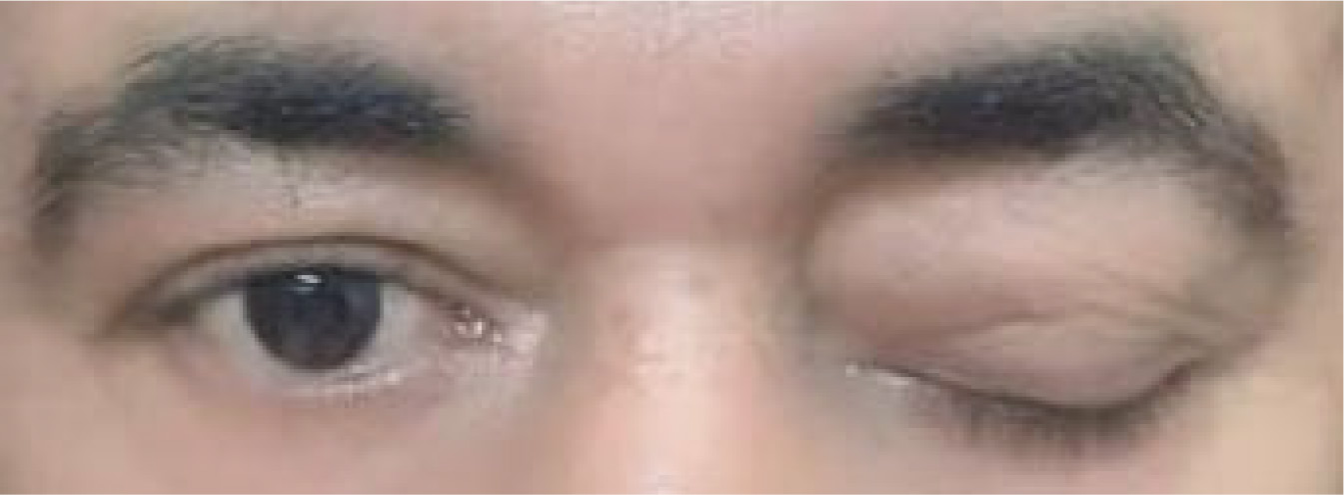
Picture of left eye ptosis.

A 43‐year‐old male diagnosed with CML 6 years ago, initially underwent a two‐year Hydroxyurea 1000 mg regimen followed by daily imatinib 400 mg. He maintained a major molecular response (MMR) of <0.1% until the onset of symptoms, including headaches and left eye ptosis, as illustrated in Figure 1, without neurological or systemic manifestations. Upon physical examination, the patient presented as a generally healthy young male with a spleen of normal size and stable vital signs.

## 3 METHODS

Various investigations during the initial assessment revealed normal liver and kidney function, but an elevated lactate dehydrogenase (LDH) level (896 U/L). His complete blood counts had a hemoglobin level of 9.1 g/dL, a white blood cell (WBC) count of 40,000 × 10^^9^/L, and a platelet count of 120 × 10^^9^/L. A peripheral blood smear revealed left‐shifted neutrophilia with 13% myeloid blasts.

Following this, bone marrow (BM) aspiration and biopsy were performed, revealed normal cellularity (65% cellularity) with 14% blasts, and immature myeloid cells. Flow cytometry analysis of BM showed 15% blast cells. These pathological findings concurred with the diagnosis of accelerated‐phase CML according to the World Health Organization (WHO) criteria of 10%–19% blasts in the BM.^16^ Polymerase chain reaction (PCR) analysis of BCR‐ABL oncogene yielded a positive result of 0.36%.

**FIGURE 2:**
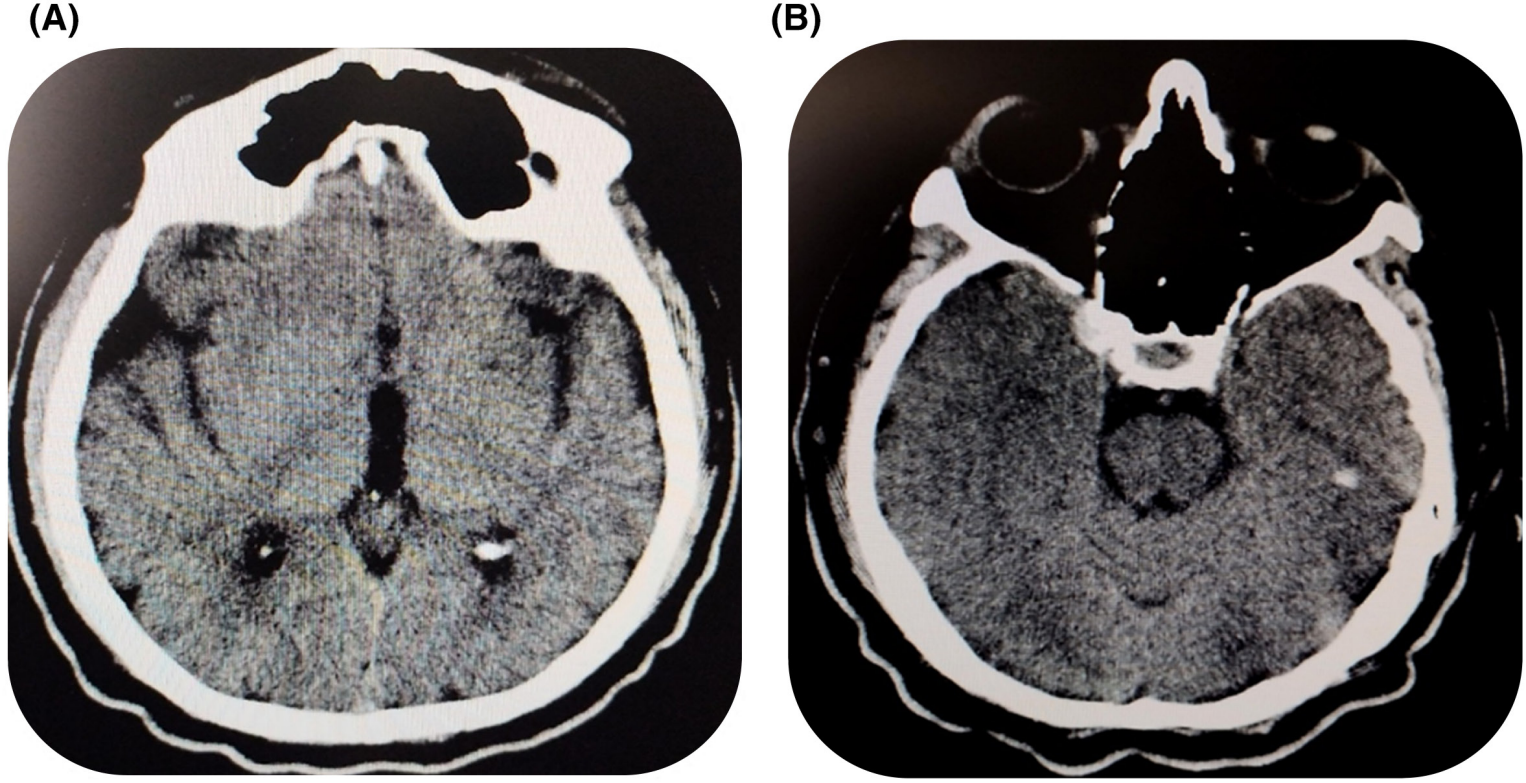
Brain CT (A, B) Multiple hypo attenuating foci are present beneath the frontal cortical subcortical regions and around the lateral ventricles on both sides. There are also multiple cystic lesions in the basal ganglia on both sides.

**FIGURE 3:**
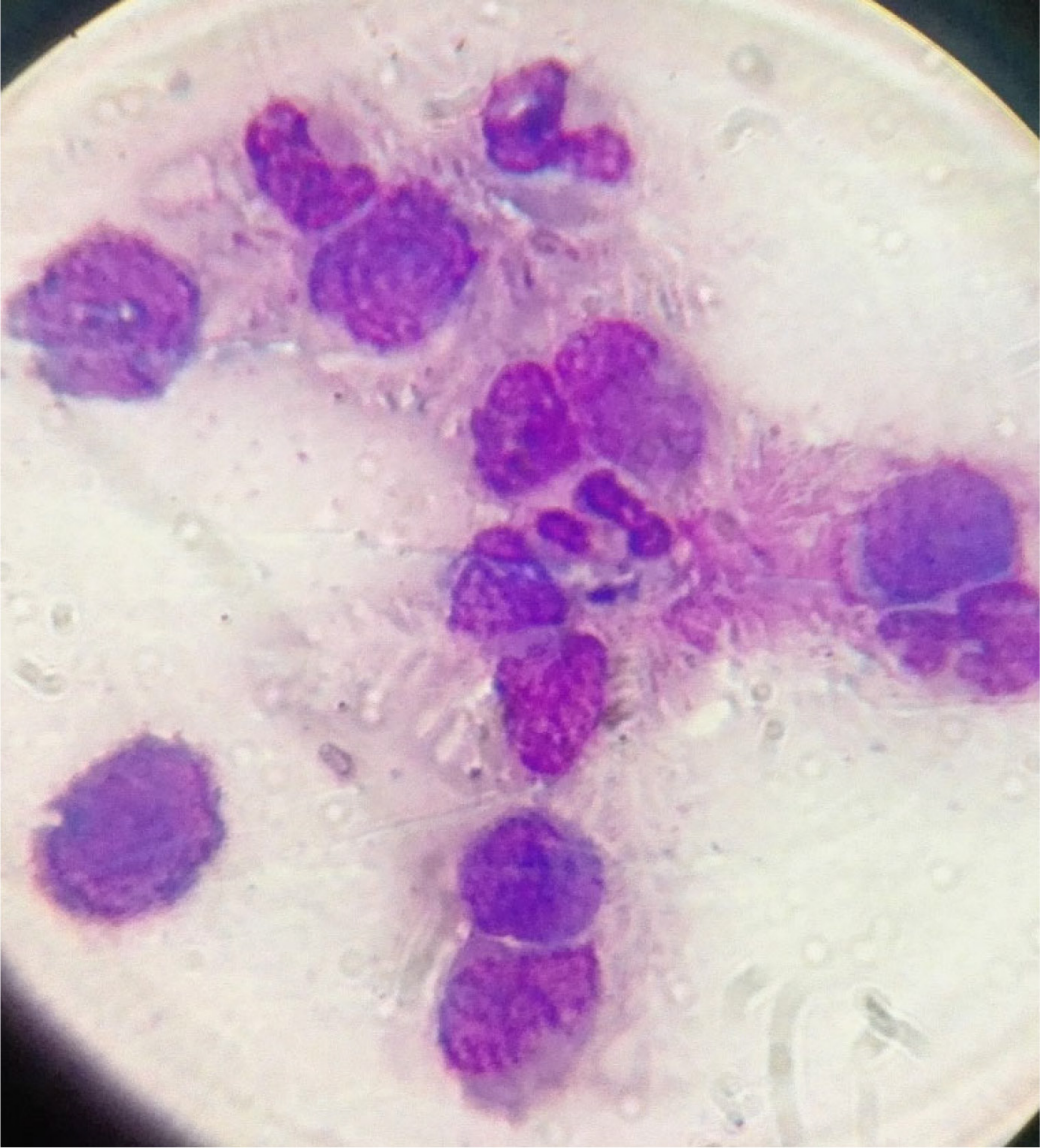
CSF examination: showed mildly cellular liquid included myeloid lineages in variable mutations and blast cells (×100 Giemsa stain's).

Computed tomography (CT) of the brain revealed numerous lesions around the cerebral ventricular area, as shown in Figure 2. Analysis of cerebrospinal fluid (CSF) via lumbar puncture (LP) revealed cloudy yellowish CSF with atypical/blast CML cells, as shown in Figure 3.

There was no history of CNS disease or indications of primary CNS malignancy, along with the absence of detected infection in the CSF. The treatment was shifted to dasatinib 100 mg once daily. Within 2 weeks, his WBC count normalized and his overall health improved. However, after 2 months, the neurological symptoms worsened. Consequently, the patient underwent a LP for CSF examination, confirming extramedullary myeloid blast infiltration in the CNS. Additionally, flow cytometry analysis of BM aspirated showed that 35% of blast cells were positive for a cluster of differentiation markers CD 13, CD 33, CD 34, CD 117, MPO, and HLA‐DR, consistent with a diagnosis of blast‐phase CML. PCR analysis of the BCR‐ABL oncogene indicated an increase in the positive result to 1.22%.

The treatment regimen was modified to maintain dasatinib at a daily dosage of 100 mg, alongside induction chemotherapy (7 + 3), consisting of cytarabine 100 mg/m^2^ and doxorubicin 45 mg/m^2^, and seven doses of intrathecal (IT) injection containing methotrexate (MTX), cytarabine, and dexamethasone were administered until disappearance of blast cells in CSF, followed by consolidation chemotherapy with cytarabine 3 g/m^2^ for 2 cycles.

## 4 RESULT AND CONCLUSION

Throughout prior treatments, the patient experienced prolonged pancytopenia and recurrent infections and had an overall poor general condition. Dasatinib was temporarily holding until recovery of the neutrophil count to ≥1.0 × 10^^9^/L and a platelet count to ≥50 × 10^^9^/L. Following this hematopoietic recovery, dasatinib was restarted at the same dosage, with regular monitoring of BCR‐ABL oncogene levels in peripheral blood by PCR and annual fluorescence in situ hybridization (FISH) testing from BM aspirate.

Despite the challenging chemotherapy, the patient remained in complete cytogenetic and molecular remission, with no evidence of active extramedullary disease for 6 months post‐diagnosis of CNS blast crisis. Unfortunately, the patient died of a severe neutropenic fever. This suggests that dasatinib may not only be effective in controlling CP CML, but also demonstrates potential in the treatment of CNS blast crisis, especially when combined with whole‐brain radiation therapy.

## 5 DISCUSSION

While CML generally presents indolently in most patients, blast crisis have a poor prognosis. Effective disease management is crucial and when feasible, proactive measures for allogeneic stem cell transplantation (allo‐SCT) should be considered.^5^ Although CNS involvement is atypical in CML, it has been well‐described.^11^ Extramedullary blast crisis typically have poor overall survival. However, our patient remained in good condition without signs of extramedullary disease relapse and survived for 6 months after CNS involvement with blast cells.

In CNS blast crisis, patients often present with clinical and radiological symptoms similar to those observed in meningitis or encephalitis. CSF testing typically reveals myeloid or lymphoblastoid cells, and molecular analysis of CSF can sometimes identify the characteristic BCR‐ABL oncogene.^7^ Despite the absence of confirmation regarding the presence of the BCR‐ABL oncogene in the CSF, conspicuous myeloid blasts were observed. Simultaneously, clear detectability of the BCR‐ABL oncogene was observed in the peripheral blood and BM.

In this case, the patient presented with remarkable clinical features, specifically left eye ptosis without concurrent neurological symptoms, alongside a left‐shifted neutrophilic. Furthermore, the absence of an enlarged spleen made it less likely to be considered a long‐standing case of CML. This unique clinical presentation presents a significant diagnostic challenge. In such clinical scenarios, a comprehensive analysis of CSF to detect atypical cells or blasts is of paramount importance. prompt initiation appropriate therapy can play a pivotal role in preventing neurological deficits.

The conventional therapeutic approaches for addressing CNS involvement in Philadelphia‐positive leukemia, such as radiotherapy, IT chemotherapy, and high‐dose systemic chemotherapy, present limitations in efficacy with transient responses.^17^ Innovative strategies are urgently needed to treat these complex cases, potentially in conjunction with treatments for primary CNS cancers, such as high‐dose MTX or temozolomide. Thus, a meticulous selection of TKIs with enhanced blood–brain barrier permeability is crucial, coupled with systemic and CNS‐directed therapy. Dasatinib, a second‐generation TKI, demonstrates superior potency in inhibiting the BCR‐ABL oncogene compared with imatinib and plays a crucial role in managing CNS CML blast crisis, particularly when combined with whole‐brain radiation therapy.^18^, ^19^, ^20^, ^21^ In our patient, dasatinib was preferred over the first‐generation TKI imatinib due to its proven ability to effectively penetrate the blood–brain barrier, inducing sustained responses in Philadelphia‐positive CNS leukemia. His treatment regimen included dasatinib administered alongside induction systemic chemotherapy complemented by IT MTX induction therapy.

In summary, it is essential to maintain a high level of vigilance when detecting CNS involvement in patients with CML receiving imatinib, particularly in patients with blast crisis. Aggressive treatment strategies include IT chemotherapy, TKI modification for better CNS penetration and even consideration of allo‐SCT.

## AUTHOR CONTRIBUTIONS

**Mais Musleh:** Conceptualization; data curation; formal analysis; funding acquisition; investigation; methodology; project administration; software; supervision; validation. **Qossay Alhussien:** Data curation; formal analysis; funding acquisition; investigation; methodology; project administration; resources; software; supervision; validation.

## FUNDING INFORMATION

No funding was required.

## CONFLICT OF INTEREST STATEMENT

The authors declare that they have no conflicts of interest.

## ETHICS STATEMENT

Not required for this case report.

## CONSENT

Written informed consent was obtained from the patient for publishing this case report and any accompanying images. A copy of the written consent is available for review by the Editor‐in‐Chief of this journal on request.

## Data Availability

Not applicable. All data (of the patient) generated during this study are included in this published article and its supplementary information files.
